# A case report of severe nasal ischemia from cold agglutinin disease and a novel treatment protocol including HBOT

**DOI:** 10.1186/s40463-019-0369-0

**Published:** 2019-10-22

**Authors:** Scott Kohlert, Laurie McLean, Dimitrios Scarvelis, Calvin Thompson

**Affiliations:** 1 ENT Associates of East Texas, 1136 E Grande Blvd, Tyler, TX USA; 2 CHRISTUS Trinity Mother Frances Health System, 800 E. Dawson, Tyler, TX USA; 3 Department of Otolaryngology – Head and Neck Surgery The Ottawa Hospital, University of Ottawa, 501 Smyth Rd, Ottawa, Ontario K1H 8L6 Canada; 4 Canada. Hyperbaric Medicine Unit The Ottawa Hospital, Ottawa, Ontario Canada; 5 Department of Medicine, University of Ottawa, Ottawa, Ontario Canada; 6 Department of Anesthesiology and Pain Medicine, University of Ottawa, Ottawa, Ontario Canada

**Keywords:** Nasal ischemia, Acrocyanosis, Cold agglutinin disease, Hyperbaric oxygen

## Abstract

Cold agglutinin disease (CAD) is a rare condition leading to blood agglutination and autoimmune hemolytic anemia. Cutaneous ischemia resulting from CAD in the head and neck is uncommon. Treatment regimens and outcomes vary widely in the literature and no clear protocol exists. This manuscript describes a patient with CAD who developed severe ischemia of the nose that resolved completely without sequellae following a medical regimen of aspirin, low molecular weight heparin, nitroglycerin ointment and hyperbaric oxygen therapy (HBOT). To our knowledge, this is the first reported case where nitroglycerin ointment or HBOT was successfully employed in the treatment of this complication.

## Introduction

Cold agglutinin disease (CAD) is a rare condition leading to blood agglutination at cold temperatures and autoimmune hemolytic anemia. The incidence of CAD is 1/1,000,000 people per year [1], and the disease accounts for 13–15% of auto-immune hemolytic anemias. Clinical manifestations can occur in colder weather and the pathologic antibodies can bind to RBC cells and cause agglutination at areas of lower body temperature (including extremities). Cutaneous manifestations of CAD include acrocyanosis [2] and Raynaud’s phenomenon [3]. Head and neck manifestations are uncommon [4–7].

Cutaneous ischemia resulting from CAD acrocyanosis in the head and neck is uncommon, with tissue loss resulting in potential disfigurement. Management and outcomes are variable, often with subsequent tissue loss (Table 1). Treatment regimens can include targeting underlying immune response, anticoagulants, and surgical debridement. We describe the successful management of this condition with vasodilators and hyperbaric oxygen therapy.

**Table 1:** Previous Case Reports of Head and Neck Cutaneous Ischemia Secondary to CAD

Authors	Involved Region(s)	Treatment Regimen	Outcome
Poldre and collagues (1985) [4]	Nasal tip, toes, fingers	Plasmapheresis, sulfinpyrazone, dipyridamole, prednisone	Loss of 8 toes, no comment regarding outcome of nasal tip ischemia
Oh and colleagues (2009) [5]	Cheek, thigh	ASA (100 mg/day), supportive wound care	Partial resolution, with some necrosis leading to permanent scarring
Jeskowiak & George (2013) [6]	Ear	Heparin, isoprost, plasmapheresis and surgical debridement	Initial improvement, subsequently lost to follow up
Mishra & colleagues (2013) [7]	Cheeks, nasal tip, ears, hands and buttocks	*Full text not available for this paper*	

## Case presentation

HC is an 84 year old female who developed a faint blue discoloration of her nasal tip following hip arthroplasty. The discoloration was initially intermittent but worsened in the postoperative period. With concerns of worsening acrocyanosis during rehabilitation, she was transferred to our institution for hematological management on postoperative day (POD) 9.

**Fig. 1:**
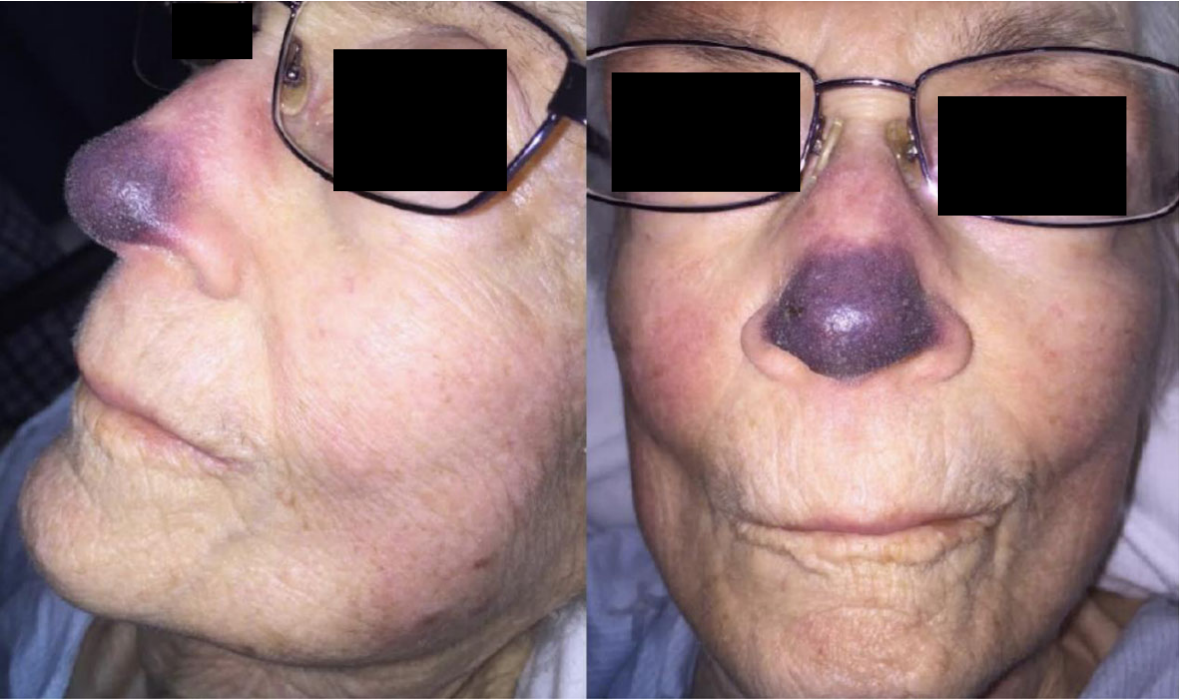
Post-admission day #1

Initial assessment revealed a violaceous plaque involving the entire nasal tip and distal dorsum (Fig. 1) with loss of sensation at the tip and pain to palpation of the skin at the periphery. There were subtle violaceous changes to the helical rims of the ears bilaterally (Fig. 1). Anterior rhinoscopy and flexible nasolaryngoscopy revealed normal nasal mucosa and no evidence of septal perforation.

Her past medical history included: primary cold agglutinin disease (CAD), severe aortic stenosis, hypertension, dyslipidemia and hypothyroidism. Medications include: Nifedipine XL, Levothyroxine, Oxazepam, Pantoloc, Pregabalin, Hydromorphone, Tramadol, Trazodone, and Warfarin. Warfarin was held and patient was on Enoxaparin in postoperative period. Family history included systemic lupus erythematosus (SLE). She was a lifelong non-smoker, with no history of recreational drug use. Her CAD had previously been treated with trials of rituximab (last dose 1 year prior to presentation) and high dose corticosteroids, although both medications had been discontinued. Prior to transfer to our institution she was given Prednisone 100 mg po × 1 dose.

The initial differential diagnosis included vasoocclusion from agglutination, vasculitis, SLE, cryoglobulinemia and anti-phospholipid antibody syndrome. Warfarin skin necrosis was considered, but found to be unlikely as it had been held prior to the arthroplasty and not restarted postoperatively.

### Laboratory investigations

Hemoglobin 75 × 10^12^/L (*ref.: 115–155*), Platelets 417 × 10^9^/L (*ref.: 130–380*). INR 1.2, PTT 28. Autoimmune workup including ESR, ANA, cANCA, pANCA, C3, C4, ENA, dsDNA, anti-cardiolipin, and lupus anticoagulant within normal limits. Mildly elevated CRP: 12.5 mg/L (*ref.: ≤ 10*). Hepatitis C serology was negative. The cold agglutinin titre was 32, with a thermal amplitude of 22 °C. The DAT was 4+ with anti-complement and negative DAT for anti-IgG. The peak titre during her disease was 128, with a thermal amplitude of 32 °C. Haptoglobin was diminished at 0.10 g/L and LDH was elevated at 288 U/ml, reticulocytes were elevated at 188.4 × 10^9^]/L, indicating ongoing hemolysis.

### Management

Two units of pRBCs were transfused on admission. No further corticosteroids were administered. Amphoteracin B was initially administered, but discontinued after 1 day as the clinical picture was not felt to be consistent with an invasive fungal infection.

**Fig. 2:**
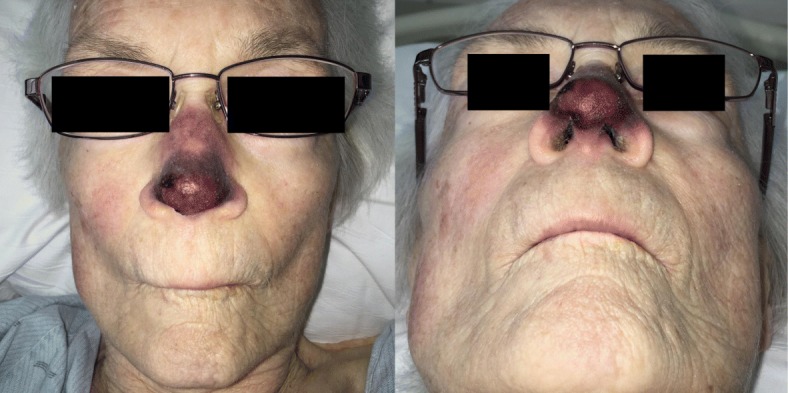
Significant worsening by post-admission day #3

The Otolaryngology and Dermatology services were consulted for assessment. They recommended to monitor and await immunological testing results. Nasal ischemia progressed acutely on post-admission day (PAD) #3, involving the entire length of the dorsum, and the tip becoming necrotic with central sloughing (Fig. 2). A midline nasal biopsy was taken at the junction between normal and ischemic tissue, revealing thrombotic vasculopathy in the superficial dermal capillaries with no surrounding inflammatory response, most consistent with thrombotic occlusion of the microvasculature of the nose secondary to agglutination.

Given the rapid clinical decline and possibility of extensive tissue loss, a multimodal management protocol consisting of nitroglycerin ointment (2% q8h), ASA (160 mg PO × 1, then 81 mg PO daily) and Enoxaparin (40 mg SC q12h until PAD#9, and then 40 mg SC daily until discharge) was initiated. Hyperbaric Oxygen Therapy (HBOT) was initiated urgently (Perry Sigma II, Dualplace Hyperbaric Oxygen Chamber, Perry Baromedical Corporation). HBOT consisted of 2.5 Atmosphere Absolute (ATA) for 90 min TID for the first 24 h, then BID for 3 days, and finally once daily for 9 days without complication. The patient underwent a total of 18 HBOT over a 2-week period.

#### Outcome and follow up

**Fig. 3:**
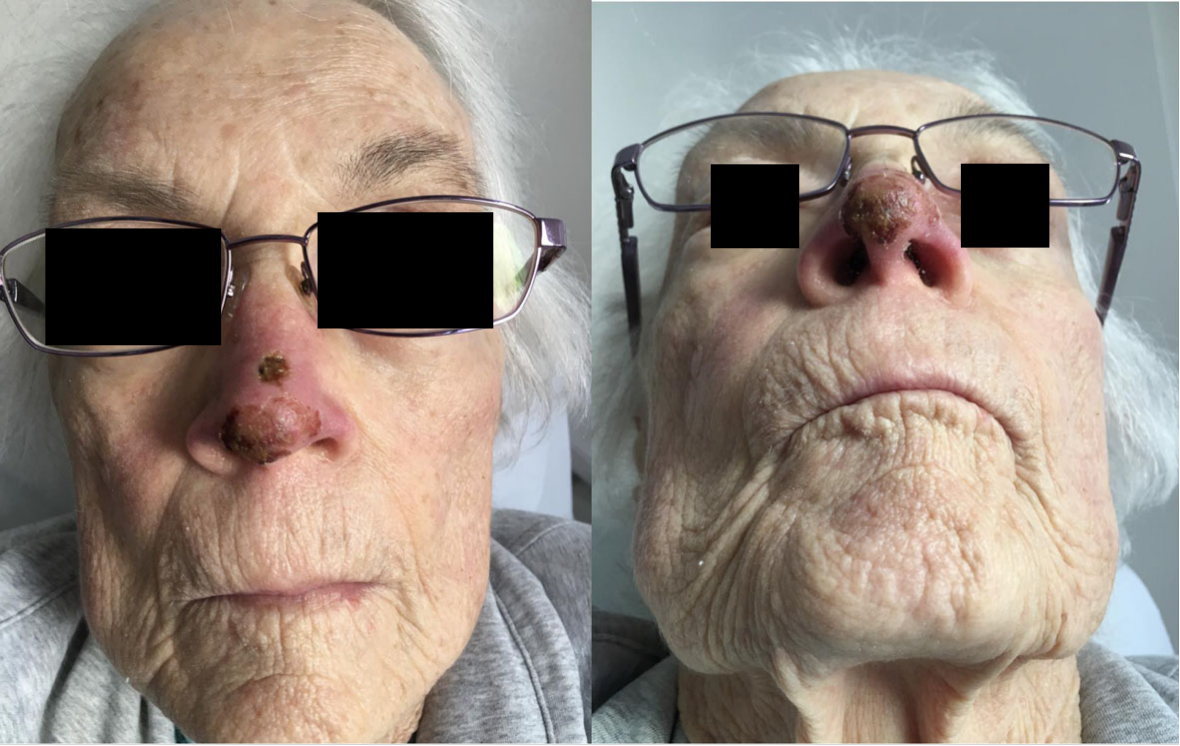
Significant improvement by post-admission day #15

**Fig. 4:**
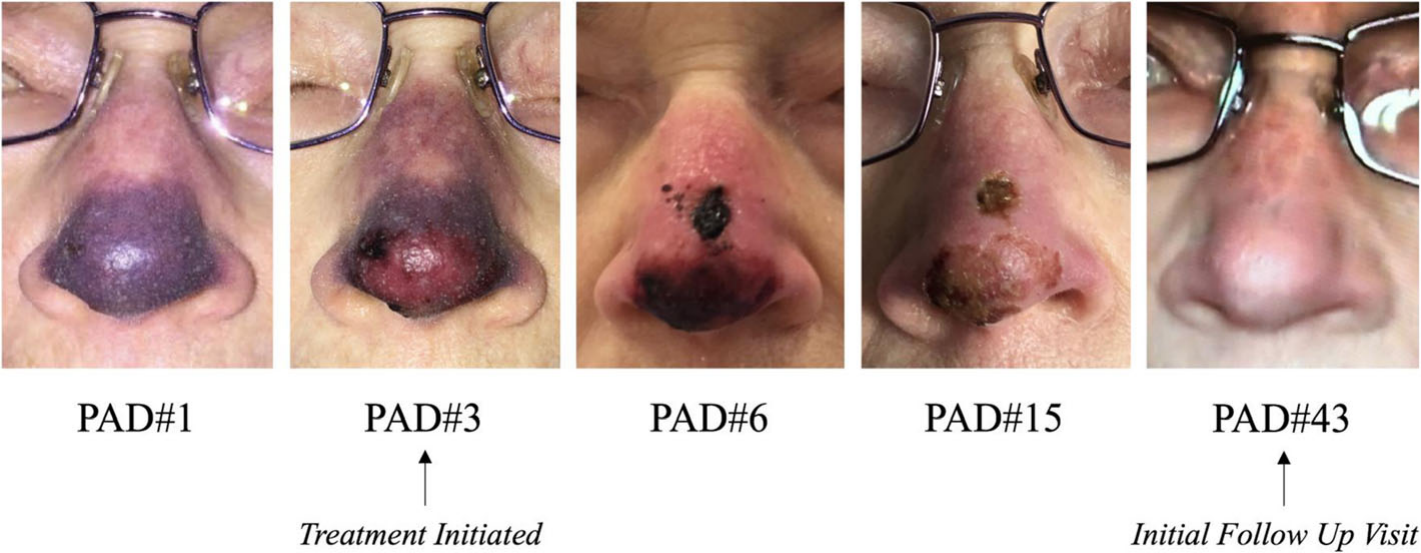
Progression of nasal ischemia throughout treatment course

The patient progressively improved (Fig. 3), and was discharged on PAD#17 with a one-month prescription for Nitro Paste BID. Prior to discharge the patient was transitioned to Warfarin which she continued to take at home. At the time of follow-up (PAD 43), had normal nasal sensation and no pain. The nose showed no evidence of ischemia. There was a faint scar at the biopsy site. The nose was no longer tender and she had regained normal sensation. Fig. 4 highlights the patient’s clinical progression throughout her treatment.

## Discussion

### Medical Management of Cold Agglutination Cutaneous Ischemia

Goals of management for cutaneous ischemia associated with CAD are to prevent further agglutination and clotting (anticoagulants, avoiding cold temperature, immunosuppressants), and optimize perfusion to ischemic tissues (vasodilators).

Heparin and ASA have both been extensively studied in the treatment of acute thrombotic disease, and have both previously been used in the management of cutaneous ischemia resulting from CAD [5, 6].

Nitroglycerin (NTG) ointment is a vasodilatory agent that has been described in the treatment of Raynaud’s syndrome [8], ischemic digital ulcers [9] and post-surgical skin flap necrosis [10, 11]. Its use has also been reported for the management of iatrogenic tissue ischemia arising from following arterial line insertion [12], inadvertent intravascular filler injection [13], and vasopressor use [14]. Potential adverse effects include headaches, light-headedness and hypotension. Careful observation with titration was necessary to avoid hypotension in setting of patient’s aortic stenosis. The use of nitroglycerin ointment has not been previously described for acrocyanosis arising from CAD.

Nifedipine is a dihydropyridine calcium channel blocker commonly used in the treatment of hypertension and coronary vasospasm (Prinzmetal’s angina). It has also been extensively studied as a treatment option for primary Raynaud’s syndrome [15], as well as for secondary Raynaud’s [16] and ischemic ulcers from scleroderma [17]. While our patient remained on Nifedipine after discharge at the recommendation of the dermatology service, the role that it played in her recovery is unclear and no overt recommendation on its use in future cases can be made.

### Hyperbaric oxygen therapy

Acute peripheral arterial insufficiency is an approved Undersea and Hyperbaric Medical Society (UHMS) indication for the use of Hyperbaric Oxygen Therapy [18]. By definition, these injuries are sudden (< 24 h), unexpected, secondary to acute arterial emboli, and exclude diabetes or venous insufficiency etiologies. The therapeutic goal common to all of these acute ischemias is to improve local tissue oxygenation and salvage tissue at risk of necrosis. HBOT is defined as inhalation of 100% oxygen in a pressurized chamber to an absolute atmospheric pressure greater than 1 ATA. Clinically, the pressure must equal or exceed 1.4 ATA in order to create a gradient that promotes adequate diffusion of oxygen from capillaries to ischemic tissue [18].

Literature to support HBOT for acute peripheral arterial ischemia is largely from case reports and series. They are difficult to interpret because of different insults or syndromes, but common in that they all result in alteration of blood flow and consequently tissue ischemia. These include traumatic arterial insufficiencies, central retinal artery occlusion, frostbite, immunologic and vasculitic disorders. Crush injuries and compromised flaps/grafts share similar features and management goals with HBOT. For acute peripheral arterial ischemia, UHMS recommends an HBOT treatment protocol of BID or TID within the first 24 h of diagnosis, followed by BID treatments until tissue at risk stabilizes, then daily until tissue fully demarcates or begins to show evidence of healing [19]. Treatment protocols are variable in the literature, often dependent timing of presentation and access to HBOT resources. Our local experience suggests HBOT is often an afterthought in management when other measures are not effective. Time to first treatment often occurs beyond 24 h from onset of symptoms. Our patient had indolent presentation over a few weeks, with later acute deterioration and involvement of entire nose. With potential for subsequent risk of loss and extensive disfigurement, urgent consideration for HBOT was sought and delivered. The exact nature of our patient’s nasal ischemia remains unclear, but was presumed secondary to autoimmune microemboli and focal ischemic necrosis.

HBOT has been used successfully following traumatic nasal tip amputation and compromised replantation and tissue salvage with adequate functional and cosmetic results [20].

### Treatment of cold agglutination

As it is felt that primary CAD is a clonal lymphoproliferative disorder, the pharmacological treatments target the presumed pathologic clonal B-cell. When testing for cold agglutinins, thermal amplitude indicates the temperature at which the antibody is active. In this case, the thermal amplitude ranged from 28°C to 32°C. These temperatures are present in the peripheral parts of the body, including the tip of the nose. Therefore, the initial treatment of CAD is the avoidance of cold environments, which is sufficient in some patients. The antibody titre of the cold reacting antibodies is less correlated to clinical manifestation of disease, as disease can be seen at titres as low as 1:32. In order to reduce the pathologic antibody-producing B-cell, pharmacological treatments including fludarabine-rituximab combination therapy have been used with variable success. Corticosteroids do not seem to have a role in treatment of CAD. In cases resistant to all therapies, chronic red cell transfusion through a blood warmer may be required [1].

## Conclusion

CAD rarely leads to acrocyanosis or cutaneous necrosis in the head and neck region. Limited data is available to guide the treatment of this condition, and no clear protocol exists. We describe a patient with CAD who developed severe acrocyanosis and ischemia of the nose that resolved completely with aspirin, LMWH, nitroglycerin ointment and HBOT.

## Data Availability

All pertinent data (including images) have been provided in the manuscript. Additional laboratory test values are available upon request.
